# Supplementary material for: Evaluation of Body Composition and Biochemical Parameters in Adult Phenylketonuria
Source: Nutrients. 2024 Oct 2;16(19):3355. doi: 10.3390/nu16193355 (PMC11478503; doi:10.3390/nu16193355)
Supplement: Supplementary file 1 [file nutrients-16-03355-s001.zip › nutrients-3234392-supplementary.pdf]

Table S1: Comparison of demographic, anthropometric and biochemical data between study and control groups

		Control Group		Study Group		p	
		Mean±SD	Median (min-max)	Mean±SD	Median (min-max)		
Age (year)		24.2 ± 4.4	23.9 (18-32.3)	24.6 ± 5.4	23.3 (18.5-41.3)	0.744	t
Gender n (%)	Male	11 42.3		24 64.8		0.076	X²
	Female	15 57.7		13 35.2			
BMI n (%)	Underweight	1 3.8		6 16.2		0.334	X²
	Normal	12 46.2		19 51.3			
	Overweight	8 30.8		7 19.0			
	Obese	5 19.2		5 13.5			
Waist circumference n (%)	Normal	20 76.9		29 78.4		0.957	X²
	Increased	5 19.2		7 18.9			
	No data	1 3.9		1 2.7			
Height (cm)		166.0 ± 8.9	165.0 (155.0-187.0)	167.7 ± 8.7	169.0 (152.0-189.0)	0.398	m
Weight (cm)		69.9 ± 14.0	67.0 (41.8-95.9)	66.5 ± 13.8	63.7 (46.3-93.8)	0.336	t
WHR		0.89 ± 0.05	0.89 (0.77-0.95)	0.87 ± 0.06	0.87 (0.74-1.07)	0.209	m
SMM percentage (%)		38.0 ± 4.9	37.2 (29.2-49.2)	39.3 ± 6.2	38.5 (26.2-49.7)	0.388	t
Fat percentage (%)		30.7 ± 8.1	32.5 (14.2-46.0)	28.2 ± 10.5	29.7 (10.0-51.3)	0.332	m
HOMA-IR n (%)	Normal	14 53.8		9 24.3		0.896	X²
	Increased	10 38.5		7 18.9			
	No data	2 7.7		21 56.8			
Glucose (mg/dl)		85.5 ± 7.3	88.5 (72-99)	84.3 ± 12.5	81 (67-123)	0.695	t
Uric acid (mg/dl)		4.6 ± 1.5	4.6 (1.8-7.7)	4.1 ± 0.8	4.2 (2.3-5.3)	0.240	t
AST (U/L)		20.0 ± 9.5	17.1 (10.7-53.7)	19.9 ± 4.5	19.5 (13.8-30.0)	0.244	m
ALT (U/L)		20.5 ± 16.0	14.5 (5.5-65.0)	20.4 ± 7.4	18.7 (9.9-41.0)	0.141	m
Total cholesterol (mg/dl)		176.9 ± 54.7	176 (21.6-307.0)	151.1 ± 31.1	168.5 (113.0-220.0)	0.207	m
Triglyceride (mg/dl)		109.4 ± 62.7	88.3 (46.5-227.3)	102.7 ± 41.2	91.0 (35-187.8)	0.936	m
HDL cholesterol (mg/dl)		50.7 ± 16.6	43.5 (21.6-86.2)	50.0 ± 13.0	48.3 (33-85.1)	0.892	m
LDL cholesterol (mg/dl)		108 ± 31.2	107 (50.0-198.0)	101 ± 29.6	104.5 (56.0-175.0)	0.409	m
Albumin (g/dl)		4.9 ± 0.2	4.9 (4.3-5.2)	5.5 ± 2.0	5.2 (4.6-15.6)	***0.001***	m
CRP (mg/L)		2.1 ± 3	0.9 (0-14.0)	7.9 ± 16.5	1.3 (0-25.0)	0.905	m
Insulin (µU/ml)		11.2 ± 6.8	9.5 (3.8-29.5)	22.0 ± 24.1	12.0 (1.1-85.9)	0.244	m
HbA1c (mmol/mol)		5.1 ± 0.4	5.1 (4.1-5.8)	4.9 ± 0.4	4.9 (4.2-5.7)	0.082	t

[t] t test / [m] Mann-whitney u test / [X²] Chi-square test

ALT: Alanine aminotransferase; AST: Aspartate aminotransferase; BMI: Body mass index; CRP: C reactive protein; HbA1c: Hemoglobin A1c; HOMA-IR: Homeostasis model assesment-insulin resistance; PKU; Phenylketonuria; SD: Standart deviation; SMM: Skeletal muscle mass; WHR: Waist to hip ratio

p<0.05 is considered to be statistically significant
